# Neurogenic thoracic outlet syndrome secondary to an unilateral cervical rib in a poodle mix

**DOI:** 10.1093/jvimsj/aalag068

**Published:** 2026-04-23

**Authors:** Madeline McDermott, Steven W Frederick, Erin Griebie, Andrew Jackson

**Affiliations:** Department of Small Animal Medicine and Surgery, BluePearl Pet Hospital, Golden Valley, MN, United States; Associate Studies Department, BluePearl Science, Tampa, FL, United States; Department of Small Animal Medicine and Surgery, BluePearl Pet Hospital, Golden Valley, MN, United States; Department of Small Animal Medicine and Surgery, BluePearl Pet Hospital, Golden Valley, MN, United States

**Keywords:** brachial plexus, dog, forelimb lameness, forelimb pain, vestigial rib

## Abstract

A 2-year-old spayed female poodle mix was evaluated for persistent intermittent right thoracic limb lameness and pain refractory to oral analgesic and anti-inflammatory medications. Orthopedic and neurologic physical examinations localized discomfort to the right shoulder and cervical region. Orthogonal right shoulder radiographs did not detect any abnormalities. Magnetic resonance imaging of the right brachial plexus revealed an anomalous bone structure at the level of the right thoracic inlet. Orthogonal cervical radiographs confirmed this finding. Surgical exploration of the right thoracic inlet identified a vestigial rib, extending from the right first rib and compressing adjacent axillary nerves, which was surgically excised via partial costectomy. This unilateral anomaly, analogous to cervical ribs described in human medicine, remains rarely documented in dogs. While rare, neurogenic thoracic outlet syndrome secondary to cervical ribs should be considered in dogs with neck pain and refractory thoracic limb lameness.

## Introduction

Cervical ribs are congenital anomalies typically arising from the cervical vertebra due to aberrant *Hox* gene expression during embryogenesis.^1^ The prevalence in humans is estimated between 0.5% and 6.2%, with many cases remaining asymptomatic or diagnosed incidentally.^2^ In contrast, cervical ribs in veterinary species, particularly dogs, are infrequently reported and even less commonly associated with clinical signs.^1^^,^^3–5^ Bilateral cervical ribs occur more frequently, though they have been described as unilateral in humans and dogs, with a preference for left-sided manifestations in unilateral cases due to incongruous somite development and expression of the *Hox* gene.^1^^,^^4^^,^^5^

Supernumerary ribs are commonly associated with thoracic outlet syndrome (TOS), brachial plexopathies, and vascular compromise.^2^ Thoracic outlet syndrome can be described as either neurogenic (brachial plexus) or vascular in nature, relating to which anatomical structures are being obstructed by the cervical rib. In humans, neurogenic TOS represents 90% of all cases, and clinical signs associated with neurogenic TOS include pain (neck, arm), paresthesia, and weakness in the adjacent muscle groups.^2^

We present a case of a young dog with a symptomatic unilateral, right-sided vestigial rib, resulting in clinical signs consistent with neurogenic TOS. This report highlights the case’s clinical relevance, diagnostic approach, and surgical management, with comparisons to analogous human disease.

## Case description

A 2-year-old, 6.2 kg spayed female poodle mix was evaluated by the primary veterinarian with an approximately 5-month history of intermittent right thoracic limb lameness and associated pain (day 0). On physical examination, the dog lifted the right thoracic limb when standing but had no obvious pain on palpation of the limb. No joint effusion or muscle atrophy was palpable. A SNAP 4Dx Plus test (IDEXX Laboratories, Westbrook, ME) was negative for *Dirofilaria immitis*, *Borrelia burgdorferi*, *Ehrlichia* spp., and *Anaplasma* spp. The dog was prescribed carprofen (2 mg/kg PO q 12 h) with initial improvement in clinical signs. However, on day 195, the dog was re-evaluated by the primary veterinarian for a 1-month history of progressive right thoracic limb lameness and a 1-week history of unwillingness to lower her head to reach the food bowl. Orthogonal radiographs of the right shoulder did not detect any abnormalities. Carprofen administration was continued, and the dog was prescribed tramadol (4 mg/kg PO q 12 h).

On day 204, the dog acutely worsened after jumping off a bed, becoming severely lethargic, anorexic, and reluctant to move, and the dog was evaluated by the emergency service at a veterinary specialty and emergency hospital. An in-house orthopedic consultation revealed pain on manipulation of the right shoulder. No neurologic deficits were noted. Gabapentin (16 mg/kg PO q 12 h) was prescribed, and carprofen and tramadol were continued. Activity restrictions were recommended. Repeated examination with an orthopedic surgeon was advised if lameness persisted.

At the surgical evaluation (day 230), the dog was doing well at home with occasional periods of pain when the right thoracic limb was palpated. Pain could not be consistently elicited on physical examination. In-house neurology consultation revealed no neurologic deficits. The dog had normal cervical range of motion with no pain noted on cervical or thoracolumbar spinal palpation. The client was given the option of a corticosteroid trial or further diagnostics including magnetic resonance imaging (MRI). A 2-week tapering course of prednisone (0.8 mg/kg PO q12 h for 7 days, then 0.8 mg/kg PO q24 h for 7 days) was prescribed following a 5-day carprofen washout period; gabapentin and tramadol were continued.

Two weeks later (day 244), the client reported persistent signs of pain. On repeated surgical evaluation (day 284), the dog exhibited caudal cervical guarding and was consistently reactive to palpation of the right shoulder region. Repeated right shoulder radiographs did not detect any abnormalities. Right brachial plexus MRI was recommended. Preanesthetic biochemistry and hematology showed mild monocytosis but were otherwise normal. Differential diagnoses included intervertebral disc disease, meningitis, neuritis, or medial shoulder instability.

**Figure 1:**
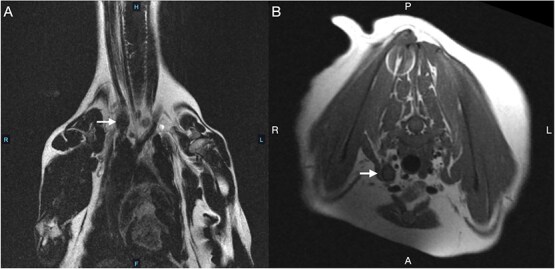
Magnetic resonance imaging of the right brachial plexus of a 6.2 kg dog with chronic, progressive right thoracic limb lameness and pain (A: dorsal T2; B: transverse T1). Findings included intervertebral disc space narrowing at C2-C3 and C3-C4 with contrast enhancement of the vertebral endplates, suggestive of disc pathology. A nodular, isointense structure near the right thoracic inlet was observed, representing an anomalous rib (arrow), with several axillary nerves and vessels identified directly adjacent to it. The right subclavian/axillary artery as it courses between the first thoracic rib and the anomalous cervical rib measured approximately 2.0 mm in diameter compared to the left subclavian/axillary artery’s diameter of 3.1 mm. At the same level, the right and left subclavian/axillary veins measured approximately 3.1 and 4.5 mm, respectively. The right and left brachial plexuses at this level measured approximately 1.5 and 2.0 mm in diameter, respectively. There was no apparent enlargement or asymmetric enhancement of the right brachial plexus adjacent to the anomalous cervical rib.

**Figure 2:**
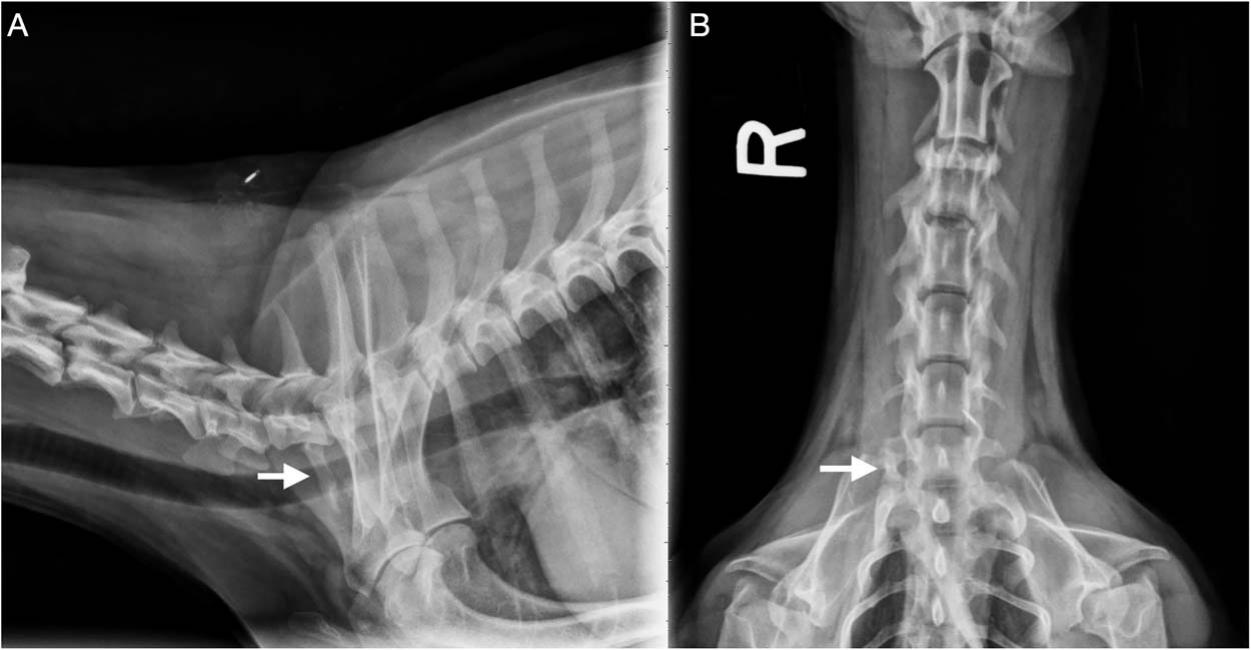
Radiographic views of the cervical spine and cranial thorax of the dog described in Figure 1 (A: lateral; B: ventrodorsal). The C2-C3 and C3-C4 intervertebral disc spaces are narrowed. There is vertebral endplate sclerosis and lucency at C3-C4 with minimal vertebral endplate changes noted at C2-C3. A right-sided malformed cervical rib is visible (arrow).

Magnetic resonance imaging (Siemens Symphony Maestro 1.5 T; Siemens Magnet Technology Limited; Oxfordshire, UK) of the right brachial plexus was performed on day 285 and revealed narrowing and loss of T2 hyperintensity of the C2-C3 and C3-C4 intervertebral discs, consistent with disc dehydration and degeneration. Mild contrast enhancement of the C2-C3 and C3-C4 vertebral endplates raised suspicion for current or prior discospondylitis. A nodular structure adjacent to the right thoracic inlet, isointense to muscle, was identified near the axillary vasculature (Figure 1). Differential diagnoses included an anomalous transitional rib, granuloma, or less likely, nerve root enlargement. Cerebrospinal fluid analysis did not detect any abnormalities. Orthogonal cervical radiographs confirmed disc space narrowing at C2-C3 and C3-C4 with endplate changes at C3-C4 and more subtly at C2-C3 and the presence of a malformed radiopaque structure on the right side (Figure 2). On palpation, marked reproducible pain was elicited directly over this structure, which was suspected to be a vestigial rib.

Based on these findings, surgical exploration of the region was pursued on day 288. An approximately 8 cm longitudinal incision was made cranial to the right scapula. Blunt dissection revealed a vestigial rib extending craniodorsally from the right first rib and compressing adjacent axillary nerves. The rib was excised via partial costectomy using rongeurs. No additional abnormalities were noted. Hemostasis was achieved using an absorbable hemostatic agent (Surgicel, Raritan, NJ). The incision was closed in 3 layers with 3-0 polydioxanone and 4-0 poliglecaprone 25 suture, and liposomal bupivacaine (0.5 mg/kg; volume expanded to 10 mL) was infiltrated into the surgical site.

Postoperative care included multimodal analgesia: buprenorphine (0.03 mg/kg TM q 8 h for 14 days), gabapentin (16 mg/kg PO q 12 h for 30 days), and robenacoxib (1.9 mg/kg PO q 24 h for 14 days). Recovery was uneventful, and the dog was discharged home the following day (day 289) with instructions to strictly limit activity. The excised rib was submitted for histopathology and was diagnosed as well-differentiated atypical cartilage proliferation and bone exostosis. There were no microscopic changes to suggest a neoplastic or infectious process.

After 2 postoperative weeks (day 299), the surgical site was well healed, and the dog showed only mild discomfort on palpation. Activity restrictions were continued for another 4 weeks, followed by a slow return to normal activity over the subsequent 4 weeks. By 10 postoperative weeks (day 361), the dog had returned to near-normal function. No further follow-up with the surgery service was pursued by the client. On day 1334, the dog was presented to the dermatology service for management of atopic dermatitis, and no orthopedic pain or lameness was reported by the client or identified on physical examination.

## Discussion

Vestigial and supernumerary ribs are rarely documented in dogs, potentially due to underreporting, and their clinical implications remain unclear.^3^^,^^6^ Pugs are more affected by cervical ribs than other breeds, with an incidence of 46% among those studied.^1^ Cervical ribs commonly originating from C7 exhibit morphologic variation supporting a hypothesized link between cervical ribs and embryologic segmentation defects.^1^^,^^6^ The dog in this case had a single, unilateral cervical rib originating from C7, with the unilateral, right-sided presentation representing a rare manifestation of this deformity.^1^

The clinical relevance of vestigial cervical ribs depends on their proximity to and interaction with adjacent structures. Compression of the brachial plexus by cervical ribs as it exits the thoracic inlet can cause neurogenic TOS, resulting in clinical signs including pain, weakness, paresthesia, and deficits in the affected limb.^2^ These clinical signs are consistent with those seen in the dog in this case. While clinical signs associated with TOS are rare, clinical signs associated with neurogenic TOS rarely improve with conservative management in people.^2^ This is consistent with the findings in this case, as the dog’s clinical signs were refractory to conservative management but resolved shortly after costectomy.

In addition to neurogenic TOS, cervical ribs can rarely result in vascular TOS when vessels are compressed or obstructed by the presence of cervical ribs. The single-case report of vascular TOS secondary to bilateral cervical ribs in a dog described small carotid vessels; though, no neurogenic clinical signs were reported in that case.^4^ There was no clinical evidence of compressive or obstructive vascular components in our case, though B wave flow ultrasound was not used to assess the vessels at the thoracic inlet.

In this case, a partial costectomy was performed to remove the cervical rib, reducing impingement of nerves exiting at C7. This was ultimately successful at reducing clinical signs associated with neurogenic TOS in the case. Partial costectomy has also been described in veterinary medicine for other structural conditions including relief of tracheal stenosis resulting from perinatal rib fracture in Japanese Black calves and relief of cervical rib-associated esophageal compression in goats.^7^^,^^8^

Diagnostic imaging modalities including radiographs, CT, ultrasound, and MRI have been described for diagnosis of cervical ribs and associated TOS.^4^^,^^5^ In this case, MRI was prioritized due to the vague clinical signs and normal shoulder radiographs, given its superior sensitivity for neurologic, soft tissue, and joint assessment. Retrospectively, early cervical radiographs reviewed by a radiologist might have expedited diagnosis and reduced cost and anesthesia time. Despite this, advanced imaging would likely still have been indicated due to the questionable clinical relevance of the cervical rib at the time of diagnosis. Owing to the limited number of reports, the optimal diagnostic protocol remains unclear.

In conclusion, this case describes a symptomatic unilateral vestigial rib directly compressing the axillary nerves in a young dog. The diagnosis was confirmed through advanced imaging and successfully treated via surgical excision of the rib. The condition parallels cervical rib syndromes seen in humans, with similar diagnostic and therapeutic strategies. Greater recognition of such anomalies might improve diagnostic accuracy and outcomes in veterinary patients evaluated for unexplained thoracic limb pain or neurogenic lameness.

## Off-label antimicrobial declaration

The authors declare no off-label use of antimicrobials.

## Institutional animal care and use committee or other approval declaration

The authors declare no institutional animal care and use committee or other approval was needed.

## Abbreviations

TOS: thoracic outlet syndrome
MRI: magnetic resonance imaging
